# The interaction between miR160 and miR165/166 in the control of leaf development and drought tolerance in *Arabidopsis*

**DOI:** 10.1038/s41598-019-39397-7

**Published:** 2019-02-26

**Authors:** Tianxiao Yang, Yongyan Wang, Sachin Teotia, Zhaohui Wang, Chaonan Shi, Huwei Sun, Yiyou Gu, Zhanhui Zhang, Guiliang Tang

**Affiliations:** 1 National Key Laboratory of Wheat and Maize Crop Science/Collaborative Innovation Center of Henan Grain Crops/College of Agronomy, Henan Agricultural University, Zhengzhou, 450002 P. R. China; 2 Department of Biological Sciences, Michigan Technological University, Houghton, Michigan 49931 USA; 3 Department of Biotechnology, Sharda University, Greater Noida, 201306 India

## Abstract

MicroRNAs (miRNAs) are a class of non-coding RNAs that play important roles in plant development and abiotic stresses. To date, studies have mainly focused on the roles of individual miRNAs, however, a few have addressed the interactions among multiple miRNAs. In this study, we investigated the interplay and regulatory circuit between miR160 and miR165/166 and its effect on leaf development and drought tolerance in *Arabidopsis* using Short Tandem Target Mimic (STTM). By crossing STTM160 *Arabidopsis* with STTM165/166, we successfully generated a double mutant of miR160 and miR165/166. The double mutant plants exhibited a series of compromised phenotypes in leaf development and drought tolerance in comparison to phenotypic alterations in the single STTM lines. RNA-seq and qRT-PCR analyses suggested that the expression levels of auxin and ABA signaling genes in the STTM-directed double mutant were compromised compared to the two single mutants. Our results also suggested that miR160-directed regulation of auxin response factors (*ARFs)* contribute to leaf development via auxin signaling genes, whereas miR165/166- mediated *HD-ZIP IIIs* regulation confers drought tolerance through ABA signaling. Our studies further indicated that *ARFs* and *HD-ZIP IIIs* may play opposite roles in the regulation of leaf development and drought tolerance that can be further applied to other crops for agronomic traits improvement.

## Introduction

MicroRNAs (miRNA) are small, endogenous, non-coding RNAs that function in gene regulation by mRNAs cleavage or translational repression in plants^1^. The target genes of most plant miRNAs encode transcription factors (TFs) and F-box proteins, which places miRNA and target genes at the center of gene regulation pathways underlying plant growth and development as well as response to biotic and abiotic stresses^2–4^. In particular, miR165/166 and miR160 are two important regulators of plant leaf development and miR165/166 also confers drought tolerance in both *Arabidopsis* and rice, through plant hormone-dependent pathways^5,6^.

In plants, few miRNA families have multiple members which target several genes. The traditional approach to understand miRNA functions is to create transgenic lines that express either miRNA-resistant targets or overexpress the miRNA genes. However, these approaches are not sufficient to decipher miRNA functions especially in case of multiple targets and misrepresentation of gene expression during miRNA overexpression^7^. Short Tandem Target Mimic (STTM), developed from Target Mimicry (TM)^8^, is an effective approach for knocking down miRNAs in plants and animals. STTMs comprises of two miRNA binding sites with a trinucleotide bulge at the potential miRNA cleavage sites, linked by a 48–88 nt spacer that can form a weak stem loop. STTM guides the degradation of small RNAs probably through the Small RNA-Degrading Nuclease (SDN) pathway^9^. This technology has been successfully employed to down-regulate numerous small RNA families in *Arabidopsis*^9,10^, rice^11,12^, tomato^13,14^ and soybean^15,16^. All of these suggest that STTM is a powerful tool for functional analysis of miRNAs in plants^17,18^.

The *Arabidopsis* genome encodes three miR160 family members (miR160a, miR160b and miR160c) with diverse functions (www.mirbase.org). The miR160 targets *AUXIN RESPONSE FACTOR (ARF)* genes, including *ARF10, ARF16 and ARF17* that also show functional redundancy^19^. *ARF10* and *ARF16* control root cap formation, while *ARF17* is involved in adventitious rooting^20–22^. *ARF10* also plays a critical role in ovary patterning, floral organ abscission and lamina outgrowth^23,24^. In contrast, the *Arabidopsis* genome encodes two miR165s (miR165a and miR165b) and seven miR166s (miR166a–miR166g) (www.mirbase.org). The mature sequences between miR165 and miR166 are nearly identical except for a C/U difference at the 17^th^ nucleotide. Class III *HOMEODOMAIN-LEUCINE ZIPPER* (*HD-ZIP III*) family genes are known targets of the miR165/166 family. In *Arabidopsis*, there are five genes that encode for the *HD-ZIP III* transcription factors, namely *PHABULOSA (PHB)*, *PHAVOLUTA (PHV)*, *REVOLUTA (REV)*, *ATHB-8* and *ATHB-15*^18,19^. Accumulating evidences have demonstrated that miR165/166 and their targets, *HD-ZIP III* genes, regulate important processes in plant development, such as shoot apical meristem (SAM) maintenance, xylem patterning and embryo formation^24–26^. Additionally, miR165/166 are also involved in the establishment of leaf polarity by repressing the expression of targets on the abaxial side of the leaf primordia^27,28^. Intriguingly, recent studies have also proven the role of miR165/166 in auxin and ABA signaling, suggesting that auxin is a regulator in miR165/166 controlled leaf development and ABA is a player in stress responses directed by miR165/166^5,10^.

Recent reports of STTM transgenics have shown remarkable developmental alterations and stress responses. The STTM160 transgenic tomatoes showed severe developmental defects, such as slender cotyledons, elongated/narrower ovaries and pear-shaped young fruits^14^. In *Arabidopsis*, STTM165/166 plants displayed multiple morphological phenotypes including twisted inflorescence stems, altered cauline branches, dark purple leaves, reduced fertility and delayed flowering. These plants also have increased indole acetic acid (IAA) contents and decreased IAA sensitivity, suggesting the possible roles of miR165/166 in auxin biosynthesis and signaling^10^. Indeed, STTM165/166 plants also displayed a drought and cold resistant phenotype and hypersensitivity to ABA during and after seed germination^5^. Over-expression of STTM166 in rice exhibited a rolled-leaf phenotype, which may be due to smaller bulliform cells and abnormal sclerenchymatous cells. The STTM166 plants also showed high drought tolerance due to reduced stomatal conductance and transpiration rates^6^. Both miR160 and miR165/166 are involved in the modulation of leaf primordium initiation, leaf polarity establishment and abiotic stress responses. Although extensive research has been concentrated on their individual roles, fewer studies have centered on their functional interactions.

In the present study, STTM160 and STTM165/166 *Arabidopsis* transgenic plants and their double mutants were generated and used to decipher their functional interactions and their specific roles in leaf development associated with auxin signaling and the ABA signaling-associated abiotic stresses. To gain a global view of their similarities and differences at the transcriptional and post-transcriptional level, RNA- and small RNA-seq technologies were applied to *Arabidopsis* STTM160, STTM165/166, and their double mutant STTM160 × STTM165/166 (STTM160 × 165/166). Our findings revealed distinct miRNA-regulatory networks between STTM160 and STTM165/166 and the interactions of these two miRNA-guided gene networks in the double mutant.

## Results

### A compromised phenotype of the double mutant compared with their parental lines

#### STTM160 × 165/166 plants displayed pleiotropic leaf development phenotypes

**Figure 1:**
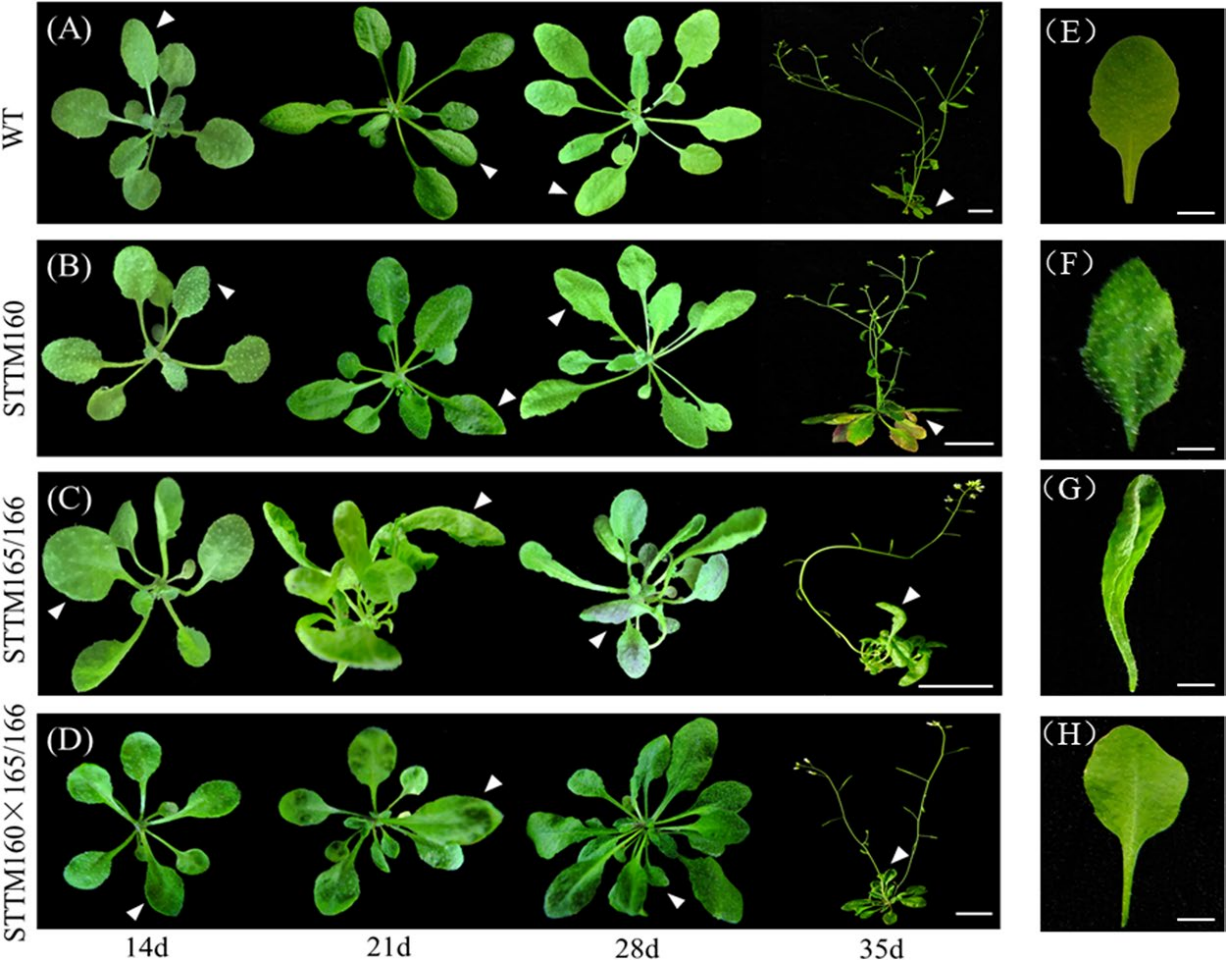
Representative whole-plant phenotypes in wild type, STTM160, STTM165/166, and STTM160 × 165/166. The typical phenotype of each genotype was observed and photographed every seven days. The genotype of plants is marked on the left. The time point for every observation is marked at the bottom. The developmental sequence is indicated within each genotype, viewed from left to right. The specific phenotype is indicated among distinct genotypes, viewed from top to bottom. (**A**) wild-type plants, (**B**) STTM160 plants, (**C**) STTM165/166 plants, (**D**) STTM160 × 165/166 plants. Data were derived from three biological replicates. The representative leaf phenotypes of (**E**) wild-type, (**F**) STTM160, (**G**) STTM165/166 and (**H**) STTM160 × 165/166.

The phenotypes of 14 to 35 days old representative individuals of the wild type, the two single mutants and their double mutants were observed. At 14-day-old stage, STTM160 showed serrated leaves, STTM165/166 displayed rounder leaves, while STTM160 × 165/166 exhibited tooth-like leaves. At 21-days STTM160 showed narrower rosette leaves with distinguished serration; STTM165/166 displayed trumpet-shaped leaves with leaflet outgrowth, while STTM160 × 165/166 plants exhibited spoon-shaped young leaves and rough mature leaves. For the 28-day-old stage, STTM160 showed more severe jagged rosette leaves, STTM165/166 displayed a dark purple color on the lower side, while STTM160 × 165/166 exhibited a dramatic increase in the number of true leaves. For the 35-day-old plants, some yellow or even purple leaves emerged in STTM160, while STTM165/166 and STTM160 × 165/166 did not change (Fig. 1A–D). We further compared the representative rosette leaf of 28-day-old plants. STTM160 showed obvious serrations, STTM165/166 displayed severe upward curled, and STTM160 × 165/166 exhibited slight downward curled rosette leaves. Thus, the rosette leaf phenotype of the double mutant is drastically weakened compared to the two single mutants (Fig. 1E–H). In addition to the leaf phenotype, other developmental phenotypes greatly varied, such as the number of siliques, the number of seeds and the flowering time (data not shown). These alterations in morphological phenotypes between two single mutants and the double mutants indicated that there may be an interaction between miR160 and miR165/166.

#### STTM160 × 165/166 plants exhibited moderate drought tolerance phenotypes

**Figure 2:**
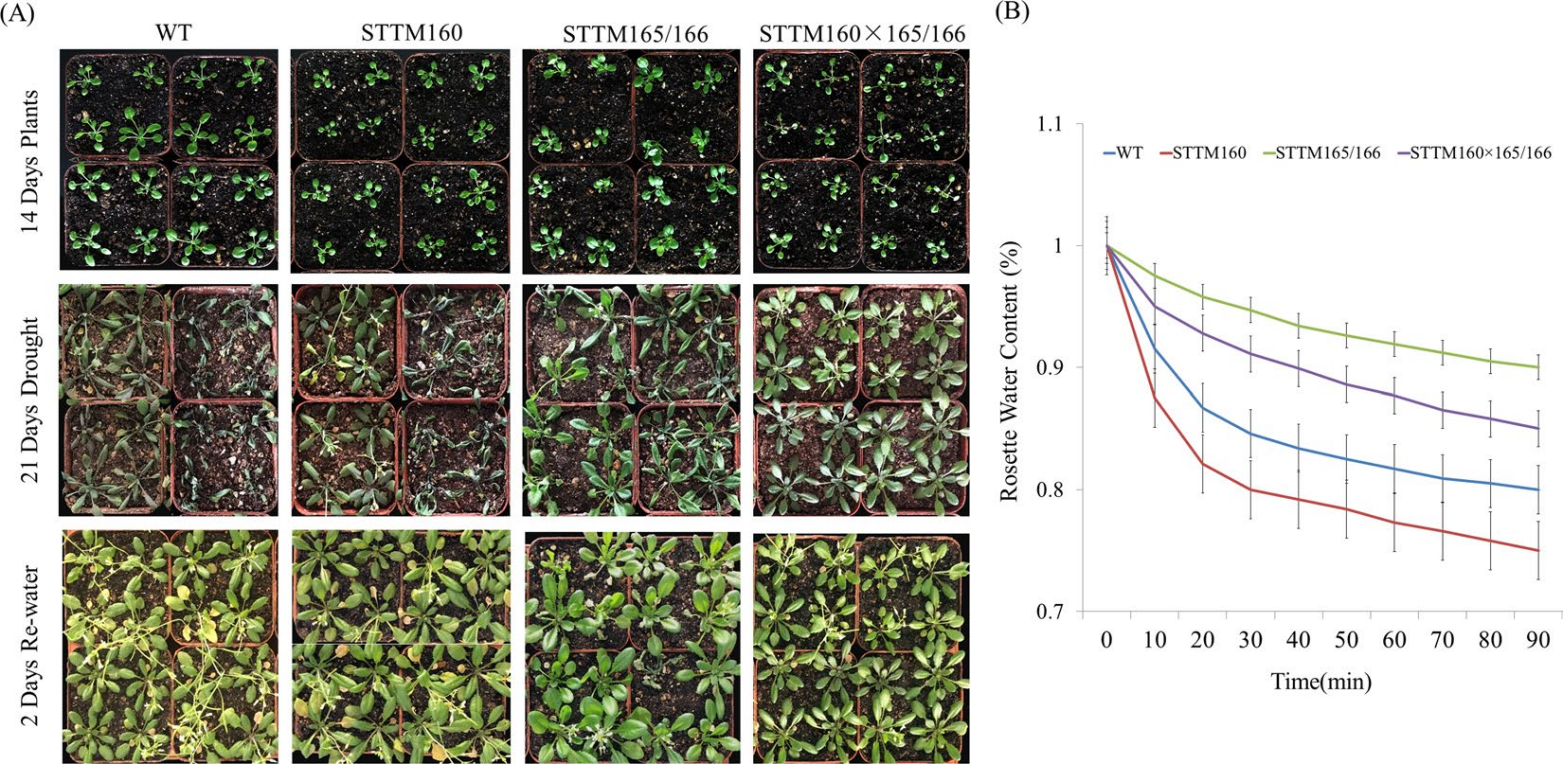
Different STTM transgenic plants displayed distinct drought tolerance characteristics. (**A**) Drought tolerance phenotype. Representative photographs show plants before drought treatment, after drought treatment, and without drought treatment. Fourteen-day-old plants were grown under well-watered condition (upper panel), after water withdrawal until 21 days (middle panel), and then re-watered for 2 days (lower panel). (**B**) Quantification of rosette leaf water content. Five rosette leaves of twenty one-day-old plants were detached and weighed at the indicated time points. Water loss at any time point was calculated as percentage of the fresh weight at time zero. Three independent experiments were performed. Bars show SE.

Seedlings were initiated under normal conditions for 14 days and the drought assay was performed by withholding water for next 21days. We then compared phenotypes of the wild type, the two single mutants and the double mutant under drought conditions. The wild type plants were severely wilted, the STTM160 plants showed moderate wilting, the STTM165/166 plants displayed a reduced growth, while the STTM160 × 165/166 plants were affected at a moderate level, showing mild wilting. The double mutants showed enhanced drought tolerance in comparison to WT and the single STTM mutants. After two days the plants undergoing drought stress were re-watered and allowed to recover and phenotypes after recovery were observed. Only a small number of WT and STTM160 plants recovered, whereas the vast majority of the STTM165/166 plants survived and recovered, and a substantial fraction of the STTM160 × 165/166 plants revived (Fig. 2A). During the drought assay, rosette leave water loss was also measured. The results indicate that the STTM165/166 and STTM160 × 165/166 plants lost water more slowly than the wild type and STTM160 plants (Fig. 2B). These differences in drought tolerance between two single mutants and the double mutants further indicated toward the possible interplay of miR160 and miR165/166.

### Differentially expressed miRNAs and genes revealed by small RNA-seq and RNA seq

#### Down regulation of target miRNAs in the double mutant and their parental lines

**Figure 3:**
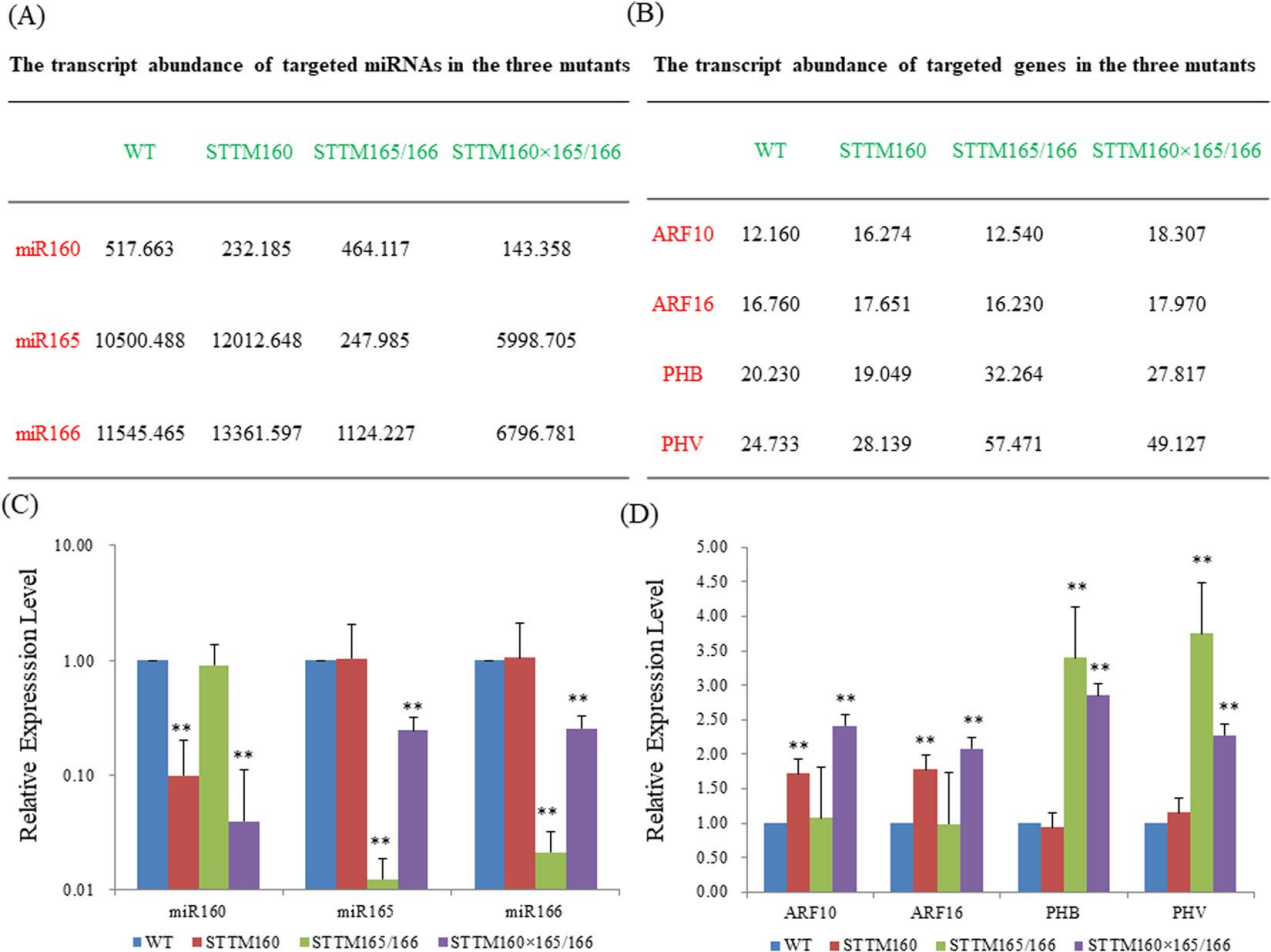
miRNA–target expression patterns between single and double mutants. (**A**) The relative expression levels of three miRNAs in two single and one double mutant as detected by small-RNA-seq. The bar in each row indicates the relative abundance of the miRNA listed on the left. Data were derived from two independent experiments. (**B**) The relative expression levels of target genes in WT and two single and their double mutant as detected by RNA-seq. Data were derived from three independent experiments. (**C**) qRT-PCR analysis of target miRNAs in double mutant compared with the two single mutants and wild type. U6 served as an endogenous control. Three independent experiments were performed, each with three replicates. Bars show SE. (**D**) qRT-PCR analysis of target genes for both miR160 and miR165/166 in double mutant compared with the two single mutants and wild type. *ACTIN2* was used as an internal control. Three independent experiments were performed, each with three replicates. Bars show SE. Asterisks in C and D indicatevalues significantly different from the wild type at P < 0.01.

We performed qRT-PCR and sRNA-seq analysis to evaluate and compare the expression level of specific miRNAs in parental mutant lines and their double mutants. As expected, the levels of miR160 and miR165/166 drastically decreased in their respective parental mutant and double mutant lines as compared with those in the wild type. The expression of miR160 was slightly reduced in STTM165/166 single mutant and that of miR165/166 slightly increased in STTM160 single mutant compared to wild type (Fig. 3A,C). The double mutant plants showed a marked decline in miR160 and  miR165/166 levels as compared to wild type (Fig. 3A,C). Notably, upon comparison of the miRNA abundance with the respective parental lines, the expression level of miR160 was found to be reduced while that of miR165/166 was enhanced in their double mutants (Fig. 3A,C).

#### Up regulation of target genes in the double mutant and their parental lines

As reduction of miRNAs levels usually causes concomittant increase in the expression of the target genes, we then measured the expression level of several target genes of miR160 and miR165/166, including *ARF10*, *ARF16*, *PHB*, and *PHV*. As expected, the expression levels of the target genes were higher in parental lines compared to that in the wild type. Intriguingly, the expression levels of *ARF10*, and *ARF16* were higher, whereas the expression level of *PHB* and *PHV* were lower in the double mutants as compared to the two single mutants (Fig. 3B,D). These results strongly proposed that STTM technology can significantly reduce the levels of specific miRNAs and concomitantly promote the expression of their target genes in transgenic plants. The differential expression pattern of few target genes, possibly resulted in a series of different leaf phenotypes.

#### The differential expression of several crucial miRNAs and their regulated downstream genes and their roles responsible for phenotypes in two single mutants and the double mutant

To dissect the molecular mechanisms of phenotypic variations mediated by STTM, we further analyzed STTM160, STTM165/166, and STTM160 × 165/166 in detail by small RNA-seq and RNA-seq. Numerous differentially expressed miRNAs and downstream genes were compared. Specifically, seven miRNAs and 728 downstream (regulated) genes were identified to be differentially expressed between STTM160 × 165/166 and STTM160, 22 miRNAs and 4732 downstream genes displayed differential expression between STTM160 × 165/166 and STTM165/166, and 24 miRNAs and 1334 downstream genes between STTM160 and STTM165/166. Upon comparisons, 37 miRNAs and 3558 downstream genes were found to be up-regulated, while 16 miRNAs and 3236 downstream genes were found to be down-regulated (Figs S1 and S2). Based on significant differences in expression, we validated six miRNAs (miR833a-5p, miR869.2, miR826a, miR5996, miR831-3p and miR8183) and their targets by using qRT-PCR. Furthermore, we also validated 12 downstream genes, including six up-regulated genes: AT1G53480, AT1G66390, AT2G31930, AT3G62710, AT3G09530, AT4G24640 and six down-regulated genes: AT1G11362, AT3G48520, AT3G16670, AT1G64160, AT1G31690, AT4G04840, respectively (Figs S3 and S4). These results showed that simultaneous inactivation of miR160 and miR165/166 altered the expression of corresponding miRNAs, thus leading to numerous possible responses, in miRNAs, in target genes or even in downstream genes.

To explore the potential roles and underlying pathways of the interaction between miR160 and miR165/166, we performed GO and KEGG enrichment analysis of all differentially expressed genes. A large number of GO terms were identified to be differentially expressed between two single and the double mutants, including response to stimulus (GO: 0050896), small molecule biosynthetic process (GO: 0044283) and response to a hormone (GO: 0009725) (Table S2). Furthermore, several pathways varied between two single and their double mutants. Within STTM160 × 165/166 and STTM160, the differentially expressed genes were suggested to be involved in photosynthesis-antenna proteins (ath00196), circadian rhythm-plant (ath04712), and starch and sucrose metabolism (ath00500). Within STTM160 × 165/166 and STTM165/166, the differentially expressed genes were indicated to participate in photosynthesis-antenna proteins (ath00196), biosynthesis of secondary metabolites (ath01110) and starch and sucrose metabolism (ath00500) (Table S3). These results demonstrated that the interaction between miR160 and miR165/166 led to great changes in diverse biochemical pathways, and of particular interest, those of plant hormone response, perception and activity.

### The interplay of miR160 and miR165/166 results in alterations in the developmental processes of leaf morphogenesis

**Figure 4:**
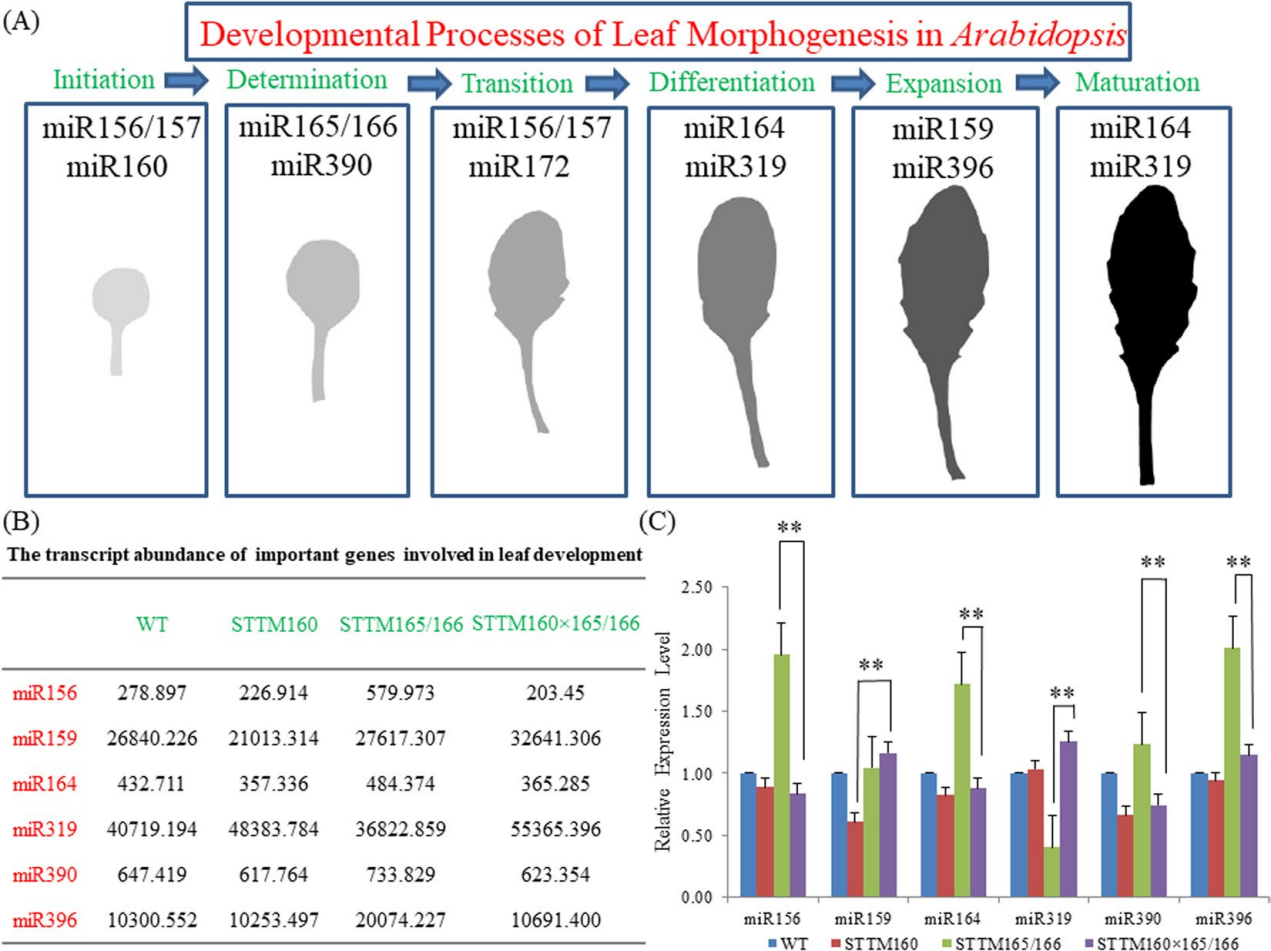
Expression patterns of some important miRNAs associated with leaf development. (**A**) A schematic pathway depicting various miRNAs that regulate the different stages of leaf development from initiation to maturation. Six main developmental processes are highlighted in blue. Corresponding miRNAs and representative leaves in the wild type are listed and shown in each box. (**B**) The relative expression abundance of several miRNAs in single- and double-mutant plants as detected by small RNA-seq. The numbers in the table refer to the normalized expression of the miRNA, each row indicates the expression abundance of the same miRNA in WT, STTM160, STTM165/166, and STTM160** × **165/166. Data were derived from two independent experiments. (**C**) The relative expression level of several miRNAs in WT, single- and double-mutant plants determined by qRT-PCR. U6 served as an internal control. Three independent experiments were performed, each with three replicates. Bars show SE. Asterisks in C indicate that values are significantly different at P < 0.01.

Since the double mutant and their parental lines showed remarkable differences in leaf phenotype, miRNAs regulating leaf growth and development were analyzed. We examined a few miRNAs known to regulate leaf morphogenesis, including miR156, miR159, miR164, miR319, miR390 and miR396 (Fig. 4A). Compared with STTM160, five miRNAs were found to be up regulated in STTM160 × 165/166 with the exception of miR156. Compared with the STTM165/166, two miRNAs (miR159, miR319) were found to be up regulated in the STTM160 × 165/166, (Fig. 4B). In particular, we found that the leaf number of STTM160 × 165/166 was much more than that of STTM160, which may be due to the higher expression of miR159. We also discovered that the leaf size of STTM160 × 165/166 was bigger than that of STTM165/166, which may due to the lower expression of miR396. Our studies also indicated that the expression of miR164 decreased but that of miR319 increased, which may further result in relatively stronger leaf serration in the STTM160 × 165/166 compared with that of STTM165/166. We also observed that the expression of miR156 and miR390 decreased in the STTM160 × 165/166, which may led to much earlier flowering in double mutants in comparison to that of STTM165/166 (Fig. 4C). Taken together, these results suggested that the interaction of miR160 and miR165/166 altered the expressions of few miRNAs toward leaf development, which affected the leaf morphology.

### The interplay of miR160 and miR165/166 results in alterations in IAA and ABA signaling pathways

**Figure 5:**
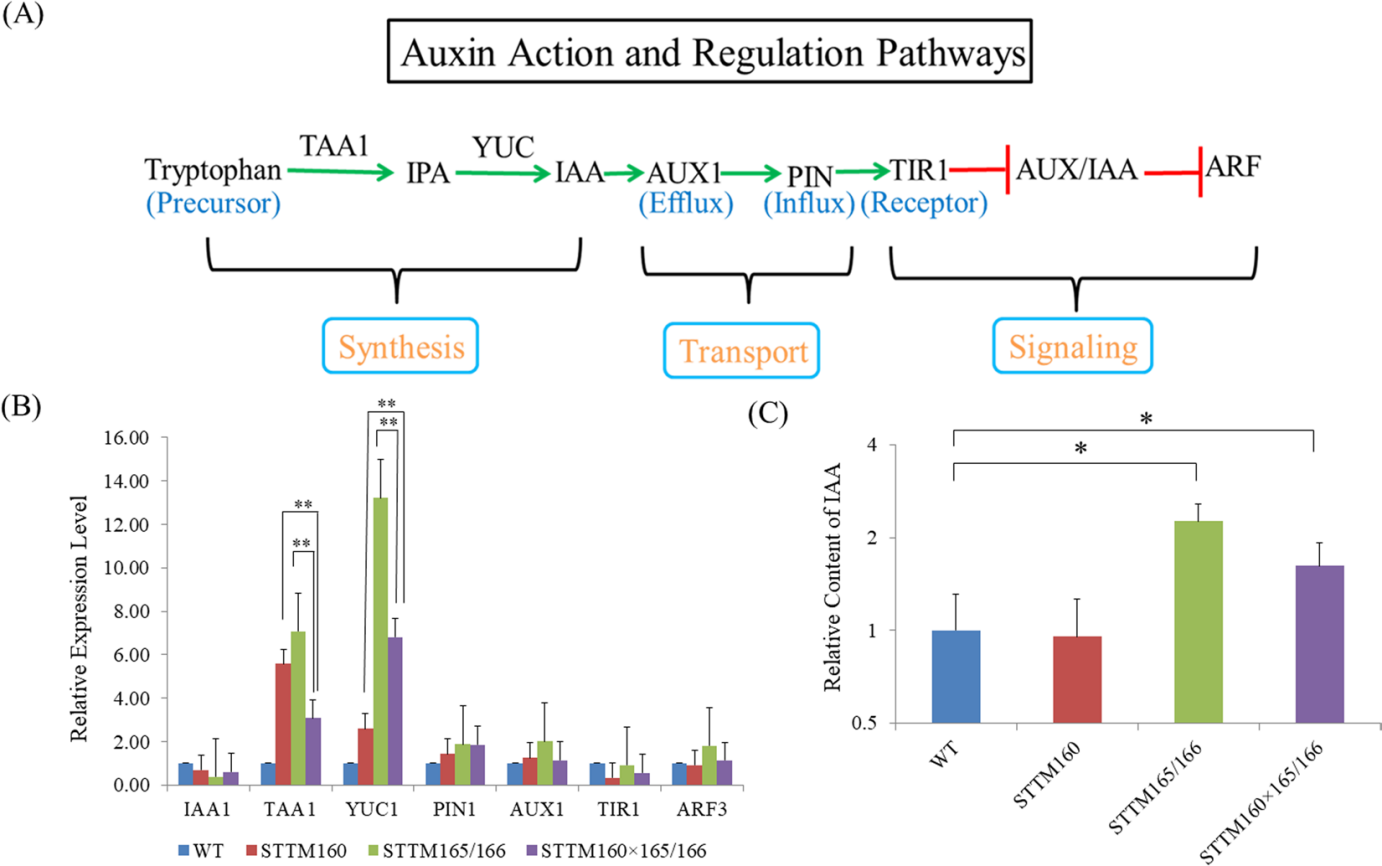
Expression patterns of some crucial genes involved in auxin biosynthesis and signaling. (**A**) IAA biosynthesis and metabolism pathway in *Arabidopsis*. There are three stages for auxin activity and regulation: synthesis, transport, and signaling. IAA biosynthesis starts from tryptophan. The formation reaction may require two critical enzymes, TAA1 and some YUCs. AUX1 is the efflux, while PIN1 is the influx carrier. These two proteins are responsible for auxin transport. TIR1 is the auxin receptor in *Arabidopsis*. The lines marked in green and red represent activated and suppressed pathways, respectively. (**B**) qRT-PCR analysis of selected genes in the wild type, two single mutants, and their double mutant. *ACTIN2* was used as an internal control. Three independent experiments were performed, each with three replicates. (**C**) The IAA content of STTM transgenic plants was calculated by HPLC. Two independent experiments were performed. Bars show SE. Asterisks in B and C indicate that values are significantly different at P < 0.01.

**Figure 6:**
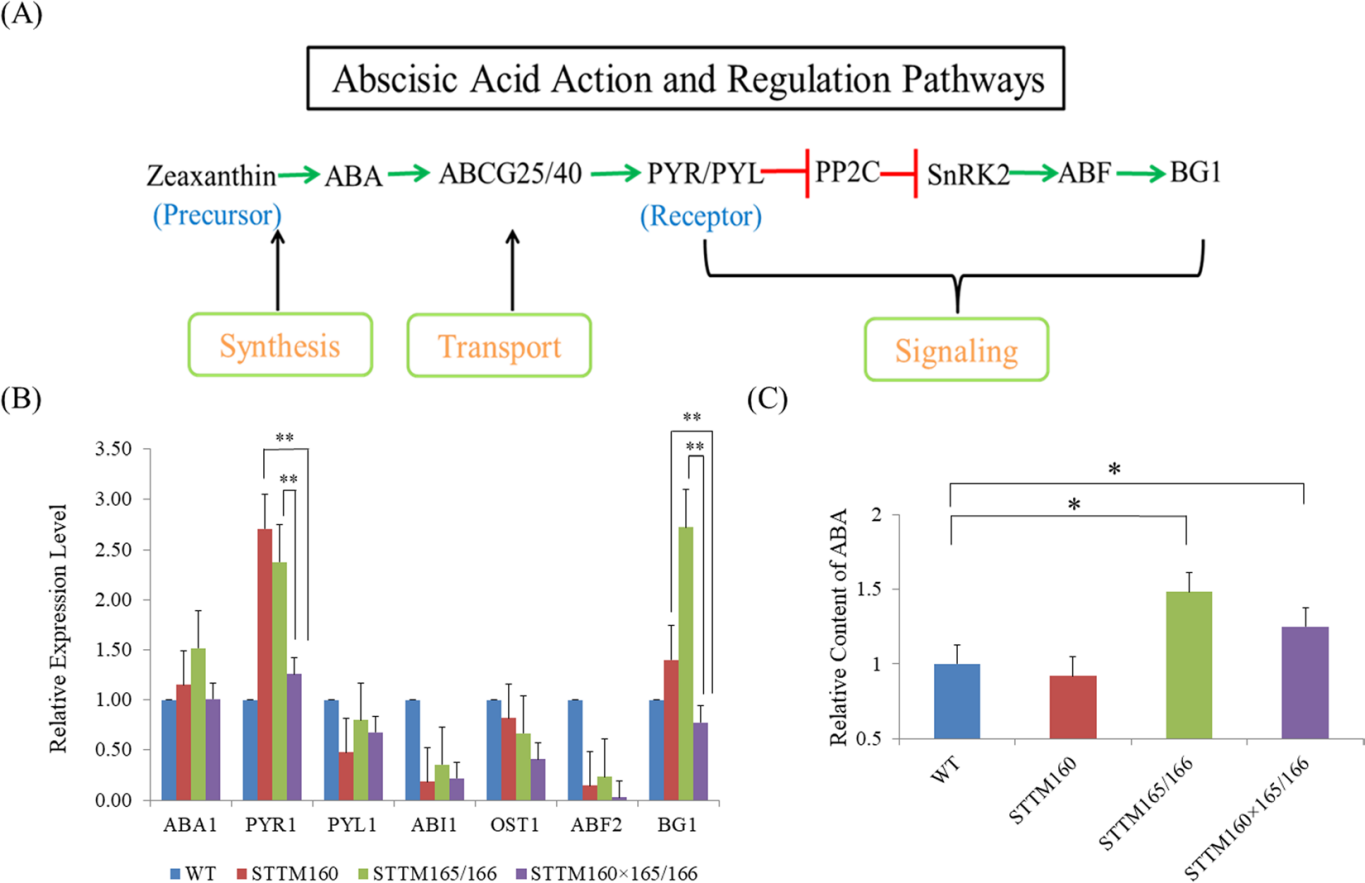
Expression patterns of some crucial genes involved in ABA biosynthesis and signaling. (**A**) ABA biosynthesis and metabolic pathway in *Arabidopsis*. The minimal set of core components for the *in vitro* reconstitution of the ABA signaling pathway includes PYR1, ABI1, OST1, and ABF2. The ABA biosynthesis is derived from zeaxanthin. PYR/PYL is the ABA receptor in *Arabidopsis*. The lines marked in green and red represent activated and suppressed pathways, respectively. (**B**) qRT-PCR analysis of selected genes in the wild type, two single mutants, and their double mutant. *ACTIN2* was used as an internal control. Three independent experiments were performed, each with three replicates. (**C**) The ABA content of STTM transgenic plants was calculated by HPLC. Two independent experiments were performed. Bars show SE. Asterisks in B and C indicate that values are significantly different at P < 0.01.

To test whether IAA and ABA signaling are involved in the crosstalk of miR160 and miR165/166, we first examined the expression of the key regulators of the IAA and ABA signaling pathways, from biogenesis to transport to signaling (Figs 5A and 6A). Our results revealed that the expression of *TRYPTOPHAN AMINOTRANSFERASE OF ARABIDOPSIS 1* (*TAA1*) was significantly lower in double mutant whereas that of *YUCCA 1* (*YUC1*) were higher in double mutant in comparison to STTM160 (Fig. 5B). In addition, the expression of *PYRABACTIN RESISTANCE 1* (*PYR1*) and *β-GLUCOSIDASE HOMOLOG 1* (*BG1*) was drastically reduced in the double mutant plants as compared with that in the two single mutants (Fig. 6B). We then investigated whether the altered expression of these key genes involved in hormone signaling could lead to any change in hormone content. As expected, the contents of IAA and ABA in STTM 160 × 165/166 were more than STTM160 but less than STTM165/166, respectively, which was found to be identical to the drought response trend (Figs 5C and 6C). These results indicated that some changes in IAA and ABA content between two single mutants and the double mutant may be due to various alterations in gene expression of the crucial genes in plant hormone signaling pathways.

## Discussion

### STTM can be used as an effective tool for functional dissection of miRNAs interactions

STTM is a highly effective and specific approach for down regulating small RNAs in *Arabidopsis*^9^. STTMs have been used to regulate the expression of miRNAs, making it possible to explore the functions of miRNAs in plants^17^. In addition to studying single miRNA functions, STTM may also be employed to dissect interactions between two distinct miRNAs. There are two major ways to achieve the above mentioned goal: one by generating a STTM line that targets two mature miRNAs, and the other by crossing two different STTM lines with each other. In our previous study, we constructed and generated the STTM319/159 transgenic lines to study the interplay of miR319 and miR159 The transgenic plants expressing single STTMs were short in height, and formed upward curled leaves and sterile flowers, which are similar to the phenotype of the double mutant plant obtained by expressing STTM319/159, together, in a single construct^17^. This phenotype is consistent with those of single MIM159 and MIM319 mutants and the double mutant obtained by crossing MIM319 and MIM159^9,29^. In the present study, we used the traditional crossing strategy to analyze the interplay of miR160 and miR165/166. As expected, the two parental lines showed multiple developmental phenotypes, including leaf morphology, flowering time and silique number. Of particular note, the double mutant integrated several developmental changes of the two single mutants. For example, leaves exhibited slightly upward growth with jagged edges, which may indicate that the leaf morphology is mediated by the interaction of miR160 and miR165/166. Other interesting phenotypes were also observed, such as moderately advanced flowering time and comparatively reduced seed number, which may further indicate that the crosstalk of these two miRNAs is directed by the compensation mechanism (Fig. 1). In addition to developmental phenotypes, the double mutants also displayed a drought tolerance phenotype, which was stronger than STTM160 but weaker than STTM165/166. We further examined the rosette leaf water loss to correlate with the drought tolerance phenotype. The water loss rate in the double mutant was very close to the STTM165/166 but much slower than the STTM160 (Fig. 2A,B). The expression of miR160 was slightly reduced in STTM165/166 single mutant but that of miR165/166 slightly increased in STTM160 single mutant compared to wild type (Fig. 3A,C). More importantly, the double mutant exhibited reduced expression of miR160 but enhanced expression of miR165/166, compared to their parental lines, respectively. This indicates toward the complexity of interaction of miR165/166 and miR160 This hypothesis was further validated in the target gene expression assay, as *ARF10* and *ARF16* were slightly enhanced, and *PHB* and *PHV* were reduced in the double mutants compared with two single mutants (Fig. 3B,D). Altogether, the above findings could provide us with new insights into the specific and overall functions of distinct miRNAs involved in plant growth and development.

### The possible model of miR160 and miR165/166 interaction in leaf development and drought tolerance

Leaf development is a complex biological process with definite decisions, directions and determinates^30,31^. Leaf development is also a sequential process that involves leaf initiation, leaf polarity, phase transition, leaf morphology and leaf senescence^32^. Small RNAs in plants, mainly miRNAs and trans-acting siRNAs (ta-siRNAs), play important roles in the control of leaf morphogenesis^33^. Drought stress is an important environmental factor that affects plant growth and development^34^. Drought stress can lead to a series of physiological and biochemical reactions in plants, including plant wilting, leaf yellowing, early flowering, increased stomatal conductance, enhanced protective enzyme activity, as well as photosynthesis and respiration inhibition^35^. Plant hormones, especially IAA and ABA, can not only regulate different tissues and organ development, but also regulate various biotic and abiotic stress responses^36–38^. Besides regulating IAA and ABA levels, miRNA have functions in abiotic stress, such as drought, cold and salt^39^.

In *Arabidopsis*, miR160 and its target genes *ARF10*, *ARF16* and *ARF17* are involved in root growth, shoot regeneration and seed germination^20–22,40,41^. miR165/166 and its target genes *PHB*, *PHV*, and *REV* are involved in meristem maintenance, root differentiation and leaf polarity formation^42–46^. In addition, miR160 and *ARFs* are involved in IAA pathways to regulate *Arabidopsis* lamina growth and soybean nodule development^23,47^. miR165/166 and *HD-ZIP IIIs* are involved in ABA pathways to regulate drought tolerance in both Arabidopsis and rice^5,6^. The individual roles of miR160 and miR165/166 have been extensively explored, however, the interactive roles between miR160 and miR165/166 is still poorly understood, especially in the control of leaf development and drought tolerance.

In this study, the double mutant of STTM160 and STTM165/166, STTM STTM160 × 165/166, was used to study the interaction of miR160 and miR165/166 in leaf development and drought tolerance in *Arabidopsis*. Firstly, the double mutant showed compromised leaf phenotypes, such as serrated young leaves and slightly upward adult leaves. This phenomenon revealed that there is a potential interaction between miR160 and miR165/166 in leaf development, and this interaction may exhibit the additive effect of two miRNAs. Moreover, extensive studies have proposed that some pivotal miRNAs are involved in leaf morphogenesis, ranging from initiation to maturation^32,33^. Thus, we examined the relative expression of these miRNAs, comprising miR156, miR159, miR164, miR319, miR390 and miR396 (Fig. 4A). We found that miR319 has the higher abundance in all plants, which may act as a pivotal factor within the whole process of leaf development (Fig. 4B,C) We also noticed that miR159 has higher abundance in the STTM160 × 165/166 compared to that in STTM160 (Fig. 4B,C). The double mutants also showed advanced flowering time, which might be explained on the basis of lower expression of miR156 in the double mutant compared to STTM165/166 single mutant (Fig. 4B,C).

Secondly, the double mutant displayed intermediate drought phenotypes. The drought tolerance of the STTM160 × 165/166 was stronger than that of the single mutant STTM160 but weaker than STTM165/166. This phenomenon further confirms the interaction between miR160 and miR165/166 in drought tolerance, and this interaction may exhibit the epistasis effect. Furthermore, previous studies have demonstrated that plant hormones, especially IAA and ABA, are associated with drought tolerance^5,10^. Thus, we identified the relative content of IAA and ABA in both single and double mutants. The relative content of IAA and ABA of the STTM160 × 165/166 was higher than that of the single mutant STTM160 but lower than STTM165/166 (Figs 5C and 6C). This result further indicates that miR160 interacts with miR165/166 under the compensation mechanism in drought tolerance.

Thirdly, we compared the expressions of some critical genes in IAA and ABA signaling pathways, and decipher the connection between miR160 and miR165/166 (Figs 5A and 6A). *TAA1* and *YUC1*, two genes responsible for IAA biogenesis, showed significant differences in the STTM160 × 165/166, compared with STTM160 and STTM165/166. *PYR1*, required for ABA transport, showed significant differences in the STTM160 × 165/166 and the single STTMs. *BG1*, critical for ABA homeostasis, also have significant differences in the STTM160 × 165/166 and the single STTMs (Figs 5B and 6B). These differentially expressed genes in the IAA and ABA signaling could be the main cause of varied IAA and ABA contents in single and double STTMs. Furthermore, previous studies have shown that miR165/166 target genes, *HD-ZIP IIIs*, can regulate expression of key genes in the IAA signaling pathway, thereby regulating the IAA regulatory network^46,48,49^. The control of this network depends on the antagonistic relationship between *HD-ZIP III* and *KANADI*, where *HD-ZIP III* activates and *KANADI* inhibits *ARFs*^50,51^. Indeed, *HD-ZIP III* can also promote *ARFs* to control *IAA* synthesis, transport, and signaling^52^. Therefore, we speculate that the interaction between miR160 and miR165/166 is established by *HD-ZIP III* promoting *ARF* expression, triggering differences in *TAA1*, *YUC1*, *PYR1* and *BG1* expression, mediating differences in IAA and ABA content, and ultimately resulting in leaf development and drought tolerance alterations.

**Figure 7:**
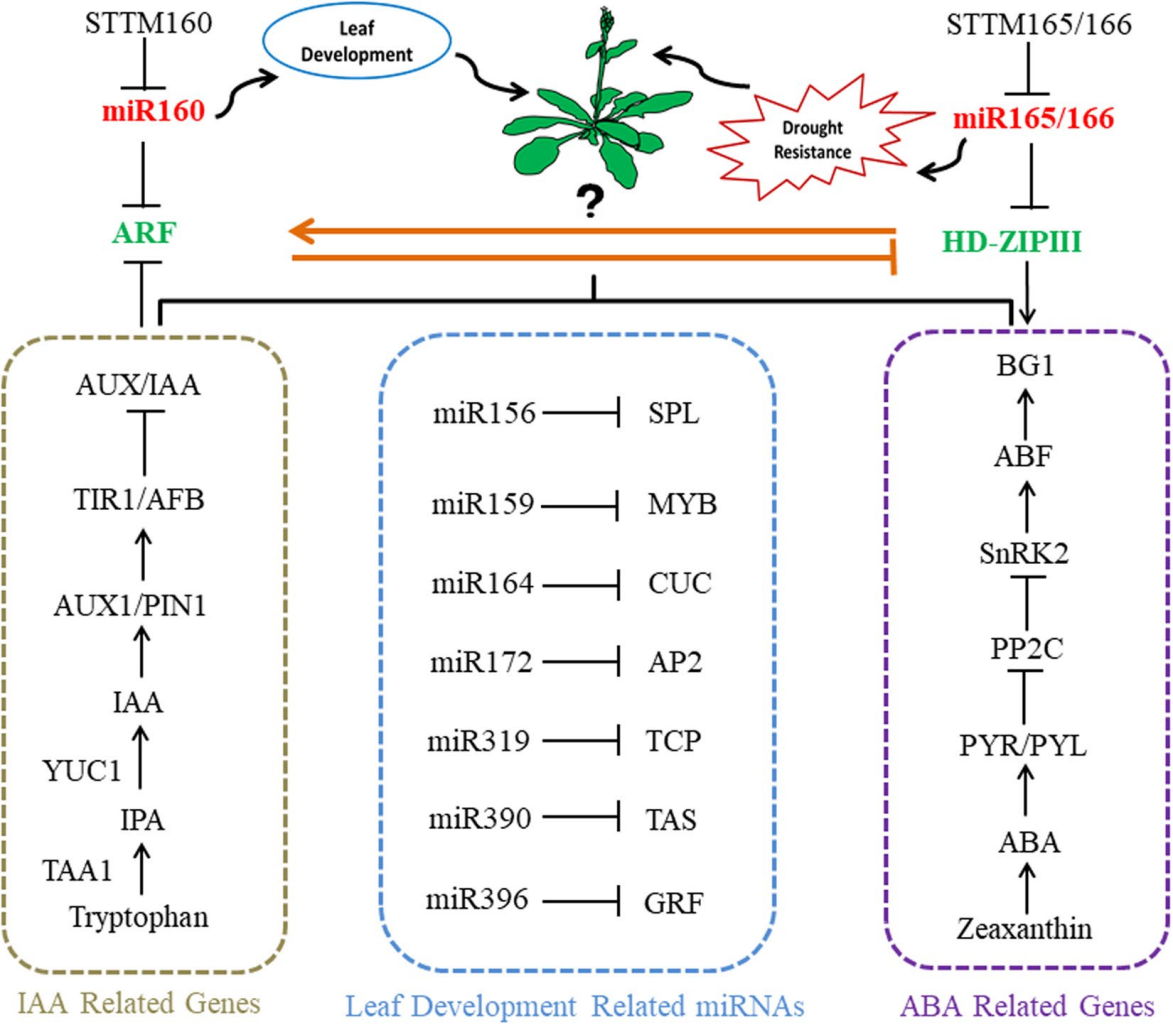
A schematic model showing the interplay of miR160 and miR165/166 regulating leaf development and drought tolerance. STTM160 effectively reduces the expression level of miR160, thus enhancing the expression level of *ARF* genes. STTM165/166 effectively reduces the expression level of miR165/166, thereby enhancing the expression level of *HD-ZIP III* genes. miR160 and its targets, *ARFs*, contribute to leaf development via the control of some important miRNAs required for the sequential processes of leaf development. miR165/166 and its targets, *HD-ZIP IIIs*, contribute to drought tolerance via the control of several critical genes required for ABA synthesis, transport and signaling. The interaction between miR160 and miR165/166 is linked by the feedback loop of *HD-ZIP III*s and *ARFs*. This study suggests that the interaction between miR160 and miR165/166 affects multiple downstream biological processes, such as auxin and ABA signal transduction and ultimately modulates leaf development and improves drought tolerance. *ARF: AUXIN RESPONSE FACTOR; HD-ZIPIII: CLASS III HOMEODOMAIN-LEUCINE ZIPPER; SPL: SQUAMOSA PROMOTER-BINDING PROTEIN-LIKE; MYB: TRANSCRITIONAL ACTIVATOR MYB; CUC: CUP-SHAPED COTYLEDON; AP2: APETALA2; TCP: TEOSINTE BRANCHED/CYCLOIDEA/PCF; TAS: TRANS-ACTING SMALL INTERFERING RNA GENE GRF: GROWTH REGULATING FACTOR; IAA: INDOLE -3-ACETIC ACID; TAA1: TRYPTOPHAN AMINOTRANSFERASE OF ARABIDOPSIS1; YUC1: YUCCA1; AUX1: AUXIN RESISTANT 1; PIN1: PIN-FORMED 1; TIR1: TRANSPORT INHIBITOR RESPONSE1;AFB: AUXIN F-BOX GENE;ABA: ABSCISIC ACID; PYR: PYRABACTIN RESISTANCE;PYL: PYRABACTIN RESISTANCE LIKE;PP2C: PROTEIN PHOSPHATASES 2C; SnRK2: SNF1-RELATED PROTEIN KINASE2; ABF: ABA RESPONSIVE FACTOR BG1: BETA-1,3-GLUCANASE 1*.

Taken together, we proposed a work model for Arabidopsis leaf development and drought tolerance mediated by miR160 and miR165/166 interactions. STTM160 represses miR160 but promotes *ARFs*. STTM165/166 represses miR165/166 but promotes *HD-ZIP IIIs*. *HD-ZIP IIIs* activate the expression of *ARF*, further evoking different expressions of leaf development-related small RNAs, such as miR156, miR164, miR319, and miR396. This interaction also trigger differential expressions of IAA and ABA signaling-related genes, such as *TAA1*, *YUC1*, *PYR1*, and *BG1*, which ultimately led to prominent variations in leaf development and drought tolerance (Fig. 7). These results not only provide useful information about miRNA interactions in *Arabidopsis* but also broaden our understanding of miRNAs functions during leaf development and drought tolerance.

### The potential application of miR160 and miR165/166 interaction in crop agronomic traits improvement

miRNAs can not only control leaf development and drought tolerance, but also regulate agronomic traits in crop plants, such as plant height, panicle branching, tillering, grain size and quality^53–57^. The purpose of improving agronomic traits can be achieved by changing the miRNA expression and their functions^58,59^. Notably, regulatinga single miRNA will improve some agronomic traits, while also exhibiting some adverse effects. For example, knock down of miR166 in rice confers drought tolerance, but also causes leaf rolling and xylem alteration^6^. Maize miR166 silenced plants showed reduced plant height, declined tassel branches and delayed flowering time compared with that of wild-type (data not shown). Indeed, miR160 and miR165/166 are two highly conserved miRNAs in both monocots and dicots. In the present study, STTM160 showed weak developmental defects, early flowering but especially bad drought tolerance. STTM165/166 showed strong developmental defects, late flowering but particularly good tolerance. STTM160 × 165/166 exhibited complementary phenotypes, improved drought tolerance and counteracted developmental defects. Thus, the double mutant displayed intermediate phenotypes that favored strong traits, while un-favoring weak traits. Because of the interaction between miR160 and miR165/166, the negative effect of miR160 silencing is offset on drought tolerance, the negative effect of miR165/166 silencing is also offset on plant height, flowering time and seed number (data not shown). In conclusion, these results provide approaches in miRNA editing to apply to field crops and provide insights in miRNA interactions to improve agronomic traits.

## Methods

### Plant materials and growth conditions

All *Arabidopsis* materials used in this study were in Columbia-0 (Col-0) background. Seeds were first sterilized with 70% (v/v) ethanol for 1 min, and then 5% (v/v) bleach for 5 min, after which they were washed with sterilized water three times. The sterilized seeds were planted on 1/2 Murashige and Skoog medium (1/2MS) (pH 5.8) containing 0.8% agar, and kept at 4 °C for 3 days for vernalization. Seedlings were transferred to sterilized soil, followed by growth in a chamber at 21 ± 1 °C under a 16 h light/8 h dark cycle. Twenty-eight-day-old plants were subsequently collected and immediately frozen in liquid nitrogen for long-term storage.

### Double mutant construction and screening

The plasmid construction of STTM160 and STTM165/166 was performed as described in our previous study^60^. The *Arabidopsis* transformation was conducted by floral dipping method^61^. In order to construct double mutants (STTM160 × 165/166), two single mutants (STTM160 and STTM165/166) were first screened. The screening was divided into two steps: first, all seeds were planted on 1/2 MS medium containing Basta. The seedlings were selected for Basta resistance. Second, the positive seedlings were transferred into sterile soil. After ten days, some leaf tissues from each positive seedling were used for PCR validation. After two rounds of selection, the positive lines of two single mutants were crossed to form the F1 generation, and then repeated sowing, screening and harvesting was done until F3 generation. The double mutant, STTM160 × 165/166, was successfully constructed for further analysis. Both single and double mutants were subjected to analysis of the expression levels of miRNAs and targets by quantitative Real Time PCR (qRT-PCR). All plant materials were photographed with a Fujifilm X-S1 digital camera (Fuji, Tokyo, Japan).

### Total RNA isolation, library construction and sequencing

The 28-day-old plant leaves of WT, STTM160, STTM165/166 and STTM 160 × 165/166 were used for total RNA isolation. Total RNA was extracted using the Trizol reagent (Invitrogen, MA, USA), and immediately monitored using 1% agarose gel electrophoresis. All RNA samples were submitted to Novogene (Beijing, China) for high throughput sequencing using the Illumina HiSeq platform.

For small RNA library construction, small RNAs, ranging from 18–30 nt, were enriched through 15% polyacrylamide gel electrophoresis, and successively ligated with 5′ and 3′ adaptors. These small RNAs were then used as templates for cDNA synthesis. Subsequently, PCR reactions, for approximately 18 cycles, were performed to acquire sufficient products for small RNA sequencing. Eight small RNA libraries (four sample tissues × two biological replicates) were constructed using the TruSeq Small RNA Sample PrepKit (Illumina Technologies, CA, USA).

For RNA library construction, mRNA was purified by using magnetic beads with oligo (dT), and immediately broken into short segments in a fragmentation buffer. Then, double stranded cDNA was synthesized and purified, followed by adaptor ligation. Next, cDNA was sorted using the AMPure XP system. Finally, the PCR was performed with the Hot Start HiFi Master Mix. 12 RNA libraries (four sample tissues × three biological replicates) were generated using the NEBNext® Ultra™ RNA Library Prep Kit for Illumina® (New England Biolabs, MA, USA).

### Bioinformatics analysis of sequencing data

The raw reads were first processed to obtain clean reads via the following steps: removing low-quality reads, trimming 5′ and 3′ adaptors, and eliminating reads containing poly (N) segments. Then, Q20, Q30 and GC contents of the clean reads were calculated.

For small RNA sequencing analysis, all clean reads, ranging from 18 to 40 nt, were mapped on the reference genome using SOAP2^62^. The expression level of miRNA was normalized using the parameter transcripts per million (TPM) in the following formula: normalized expression = (actual miRNA count/total count of clean reads) × 10^6^. The differentially expressed miRNA was defined using the combined criteria of fold changes and P-values [fold change = log2 (normalized read counts of treated group/control group), P-value was calculated using Pearson’s chi-square test]. We also used the psRNATarget server with its default parameters for prediction of the putative targets of *Arabidopsis* miRNAs^63^.

For RNA sequencing analysis, all clean reads with paired-ends were mapped using TopHat2^64^. The expected number of fragments per kilobase of transcript sequence per million base pairs sequenced (FPKM) was used for normalization. Based on the negative binomial distribution, a method called DESeq was employed for differential expression analysis^65^. Genes with an adjusted P-value < 0.05 detected by DESeq were regarded as differentially expressed. Moreover, to annotate the function of interest genes, two online resources were used: the Gene Ontology (http://www.geneontology.org/) and the Kyoto Encyclopedia of Genes and Genomes (http://www.genome.jp/kegg/).

### Quantitative real-time PCR (qRT-PCR)

Total RNA was isolated from leaves using the Trizol reagent (Invitrogen, MA, USA) in accordance with the manufacturer’s protocol. For normal qRT-PCR, the total RNA (about 1 μg) was reverse-transcribed using the PrimeScript™ RT reagent kit with gDNA Eraser (Perfect Real Time), and then the target gene expression was quantified using the SYBR® Premix EX Taq™ II (Tli RNaseH Plus) Kit (Takara, Dalian, China). For poly (A) tailing qRT-PCR, the Mir-X™ miRNA qRT-PCR SYBR® Kit (Takara, Dalian, China) was used to evaluate the expression levels of selected miRNAs. The qRT-PCR reactions were performed on the CFX96 Touch™ Real-Time PCR Detection System (Bio-Rad, CA, USA). *ACTIN2* and U6 snRNA were used as internal controls for normal qRT-PCR and poly (A) tailing qRT-PCR, respectively. The ΔΔCt method was carried out to measure relative expression levels^66^. Three independent experiments were performed, each with three biological replicates. All primers used for qRT–PCR are listed (Table S1).

### Drought stress treatment and plant hormone measurement

All seeds were sowed directly in soil and then grown in a chamber under long day conditions (16 h light/8 h dark cycle). Seven days after sowing, the seedlings were transplanted into new pots with same weight of sterilized soil. At 14 days after sowing, drought treatment was performed via withholding water for 21 days. After two days, stressed plants were re-watered and the survival phenotypes were observed. The positions of pots were rotated every other day to minimize the effect of the environment. For rosette water loss, rosette leaves of 21-day-old plants were cut from the base and weighed at the indicated time points. Three independent experiments were performed, each with three biological replicates. The water loss rate was calculated as the percentage of initial fresh weight. For soil water content, each pot was weighed every day during the drought stress treatment. Soil water content percentage was calculated as the percentage of initial pot weight. Three independent experiments were performed, each with three biological replicates.

The aerial parts for 28-day-old plants of WT, STTM160, STTM165/166 and STTM160 × 165/166 were collected and immediately frozen in liquid nitrogen for phytohormone quantification. All samples were grinded in the 5 ml extraction solution of 80% (v/v) ethanol and 1% (w/v) polyvinylpyrrolidone-40. After 90 min of incubation, the extract was centrifuged at 20000 g for 15 min at 4 °C. The supernatant was filtered through Sep-Pak Plus C18 (Waters, Milford, MA, USA) and then evaporated at 40 °C under vacuum. The organic phases containing IAA and ABA were completely dried under vacuum, dissolved in 300 µL of 100% methanol, and stored at −70 °C. All samples were submitted to Comin (Suzhou, China) for IAA and ABA content measurement using the High Performance Liquid Chromatography (HPLC) system. The standard samples of IAA and ABA were from Sigma-Aldrich (St. Louis, MO, USA). Four independent experiments were performed, each with three biological replicates.

## Supplementary information

Supplementary Material

Dataset 1

Dataset 2

Dataset 3

## Data Availability

All data generated or analyzed during this study are included in this published article (including its Supplementary Information Files).
