# Supplementary material for: The interaction between miR160 and miR165/166 in the control of leaf development and drought tolerance in Arabidopsis
Source: Sci Rep. 2019 Feb 26;9:2832. doi: 10.1038/s41598-019-39397-7 (PMC6391385; doi:10.1038/s41598-019-39397-7)
Supplement: Supplementary file 1 — Supplementary Material [file 41598_2019_39397_MOESM1_ESM.pdf]

The interaction between miR160 and miR165/166 in the control of leaf development and drought tolerance in *Arabidopsis*

Tianxiao Yang[1,2 *], Yongyan Wang[1,2*], Sachin Teotia[1,2,3], Zhaohui Wang[1], Chaonan Shi[1], Huwei Sun[1], Yiyou Gu[2], , Zhanhui Zhang[1,†] , Guiliang Tang[1, 2 †]

[1] National Key Laboratory of Wheat and Maize Crop Science/Collaborative Innovation Center of Henan Grain Crops /College of Agronomy, Henan Agricultural University, Zhengzhou 450002, P.R. China
[2] Department of Biological Sciences, Michigan Technological University, Houghton, Michigan 49931, USA
[3] Department of Biotechnology, Sharda University, Greater Noida, 201306, India

Guiliang Tang
[1] Collaborative Innovation Center of Henan Grain Crops/National Key Laboratory of Wheat and Maize Crop Science/College of Agronomy, Henan Agricultural University, Zhengzhou 450002, P.R. China
[2] Department of Biological Sciences, Michigan Technological University, Houghton, Michigan 49931, USA
E-mail: gtang1@mtu.edu
Phone: 906-487-2174

Zhanhui Zhang
[1] Collaborative Innovation Center of Henan Grain Crops/National Key Laboratory of Wheat and Maize Crop Science/College of Agronomy, Henan Agricultural University, Zhengzhou 450002, P.R. China
E-mail: amair0534@163.com
Phone: 86-371-63558122

Supplementary Material

This file includes Supplemental Figures 1-4 and Supplemental Table 1-3 (attached separately as 3 Excel files).

Supplementary materials are available at Scientific Reports online.

Figure S1. Differentially expressed miRNAs and genes among STTM160, STTM165/166 and STTM160×165/166 shown by heat map.

Figure S2. Differentially expressed miRNAs and genes among STTM160, STTM165/166 and STTM160×165/166 shown by Venn diagram.

Figure S3. qRT-PCR validation of differentially expressed miRNAs from small RNA seq and their targets.

Figure S4. qRT-PCR validation of differentially expressed genes from RNA seq.

Table S1. All primers used for qRT-PCR in this study.

Table S2. GO analysis of all differentially expressed genes from RNA sequencing.

Table S3. KEGG analysis of all differentially expressed genes from RNA sequencing.

Supplementary Figure Legends

Figure S1. Differentially expressed miRNAs (A), and genes (B), among STTM160, STTM165/166 and STTM160×165/166 shown by heat map. The red color indicates up-regulated, while blue indicates down-regulated in single mutants compared to the double mutant.

Figure S2. Differentially expressed miRNAs (A), and genes (B), among STTM160, STTM165/166 and STTM160×165/166 shown by Venn diagram. SG1, STTM160; SG2, STTM160; DB, STTM160×165/166

Figure S3. qRT-PCR validation of a few differentially expressed significant miRNAs (labeled in red) from small RNA sequencing and their targets (labeled in green). First Column, STTM165/166 *VS* STTM160; Second Column, STTM160 *VS* STTM160×165/166; Third Column, STTM165/166 *VS* STTM160×165/166.

Figure S4. qRT-PCR validation of a few differentially expressed significant genes from RNA sequencing. Genes labeled in red indicate up-regulated, while those labeled in green indicate down-regulated. First Column, STTM165/166 *VS* STTM160; Second Column, STTM160 *VS* STTM160×165/166; Third Column, STTM165/166 *VS* STTM160×165/166.

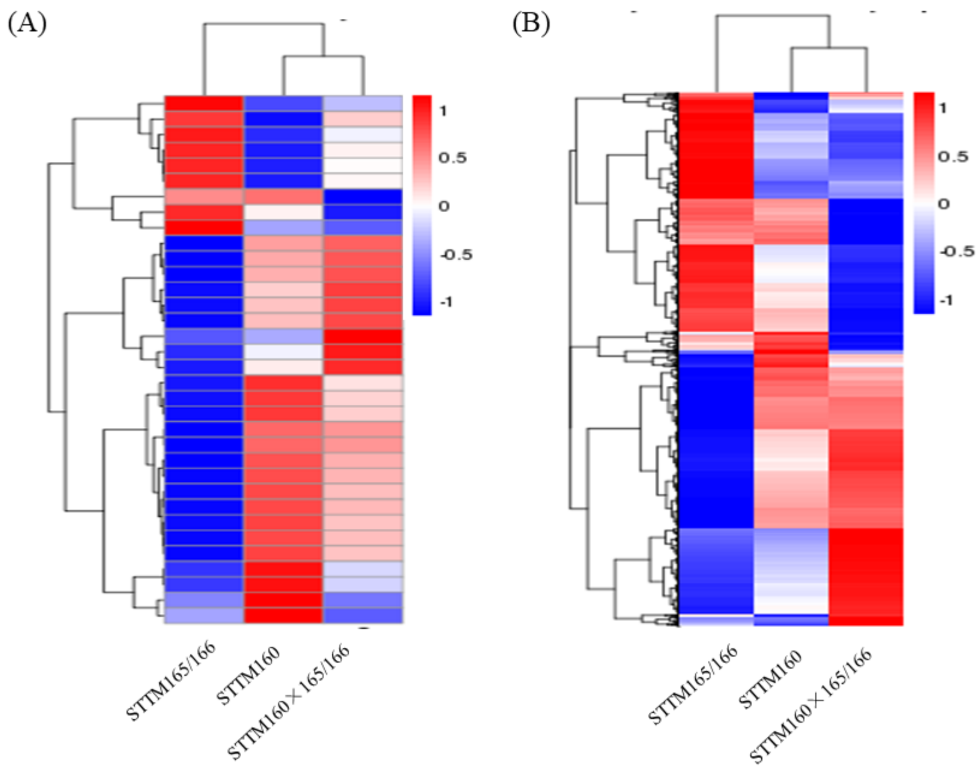

Figure S1. Differentially expressed miRNAs (A), and genes (B), among STTM160, STTM165/166 and STTM160×165/166 shown by heat map. The red color indicates up-regulated, while blue indicates down-regulated in single mutants compared to the double mutant.

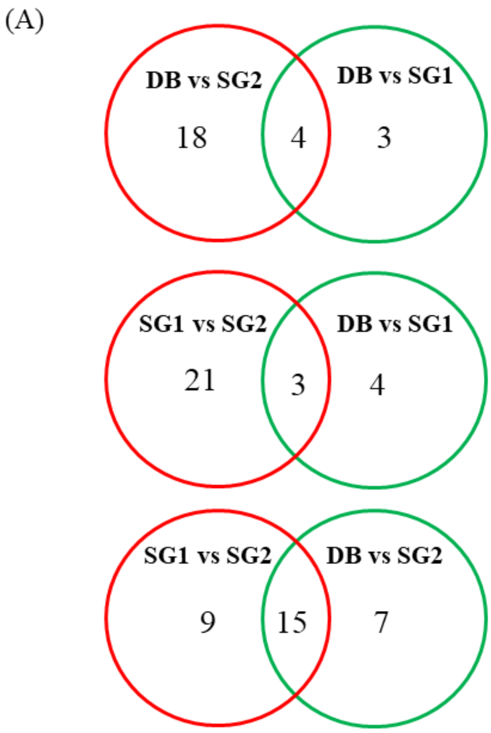

Figure S2. Differentially expressed miRNAs (A), and genes (B), among STTM160, STTM165/166 and STTM160×165/166 shown by Venn diagram. SG1, STTM160; SG2, STTM160; DB, STTM160×165/166

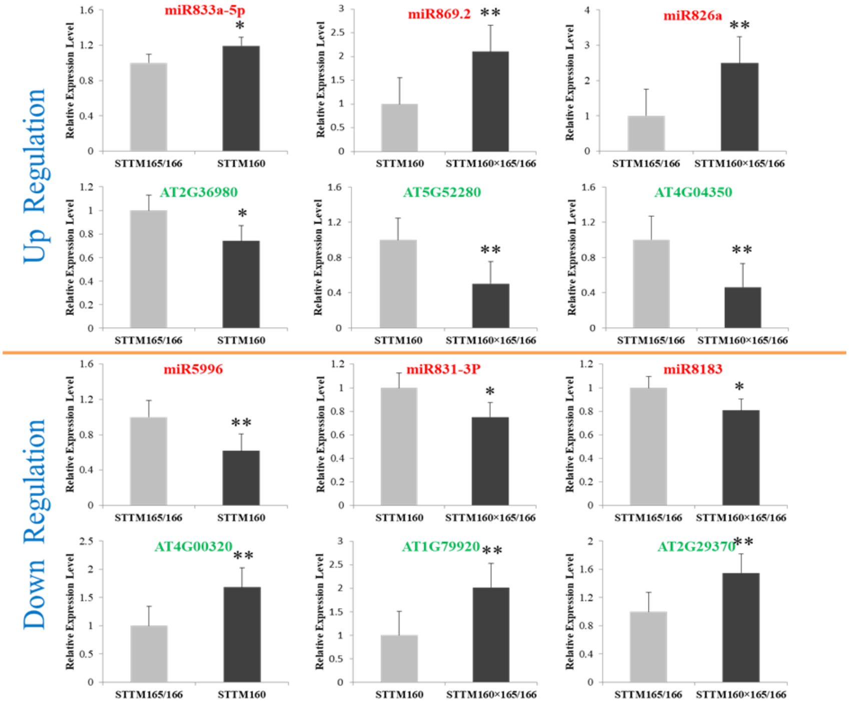

Figure S3. qRT-PCR validation of a few differentially expressed significant miRNAs (labeled in red) from small RNA sequencing and their targets (labeled in green). First Column, STTM165/166 *VS* STTM160; Second Column, STTM160 *VS* STTM160×165/166; Third Column, STTM165/166 *VS* STTM160×165/166.

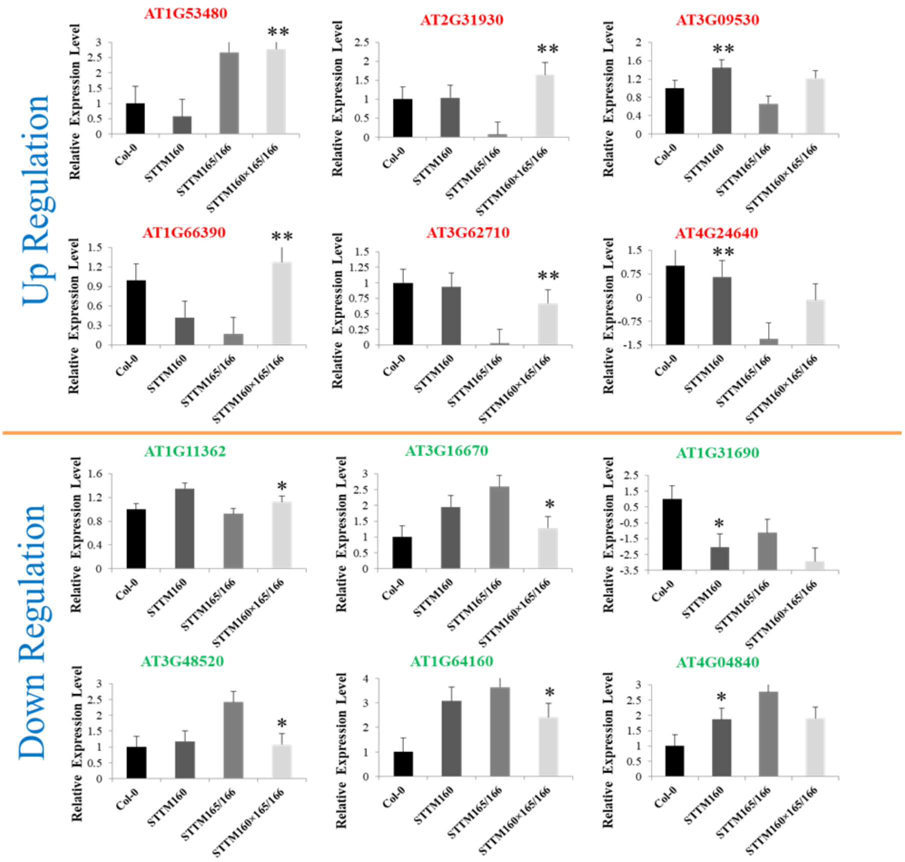

Figure S4. qRT-PCR validation of a few differentially expressed significant genes from RNA sequencing. Genes labeled in red indicate up-regulated, while those labeled in green indicate down-regulated. First Column, STTM165/166 *VS* STTM160; Second Column, STTM160 *VS* STTM160×165/166; Third Column, STTM165/166 *VS* STTM160×165/166.
